# Yeast karyopherin Kap95 is required for cell cycle progression at Start

**DOI:** 10.1186/1471-2121-11-47

**Published:** 2010-06-29

**Authors:** Francisco José Taberner, Juan Carlos Igual

**Affiliations:** 1 Departament de Bioquímica i Biologia Molecular, Universitat de València. Dr. Moliner 50, 46100 Burjassot (Valencia), Spain

## Abstract

**Background:**

The control of the subcellular localization of cell cycle regulators has emerged as a crucial mechanism in cell division regulation. The active transport of proteins between the nucleus and the cytoplasm is mediated by the transport receptors of the β-karyopherin family. In this work we characterized the terminal phenotype of a mutant strain in β-karyopherin Kap95, a component of the classical nuclear import pathway.

**Results:**

When *KAP95 *was inactivated, most cells arrested at the G2/M phase of the cell cycle, which is in agreement with the results observed in mutants in the other components of this pathway. However, a number of cells accumulate at G1, suggesting a novel role of Kap95 and the classical import pathway at Start. We investigated the localization of Start transcription factors. It is known that Swi6 contains a classical NLS that interacts with importin α. Here we show that the *in vivo *nuclear import of Swi6 depends on Kap95. For Swi4, we identified a functional NLS between amino acids 371 and 376 that is sufficient and necessary for Swi4 to enter the nucleus. The nuclear import driven by this NLS is mediated by karyopherins Kap95 and Srp1. Inactivation of Kap95 also produces a dramatic change in the localization of Mbp1 since the protein is mainly detected in the cytoplasm. Two functionally redundant Kap95- and Srp1-dependent NLSs were identified in Mbp1 between amino acids 27-30 and 166-181. Nuclear accumulation was not completely abolished in a *kap95 *mutant or in the Mbp1 mutated in the two NLSs, suggesting that alternative pathways might contribute to the Mbp1 nuclear import to a lesser extent.

**Conclusions:**

Kap95 plays an essential role at the initiation of the cell cycle by driving the nuclear import of Swi4, Swi6 and Mbp1, the three transcription factors responsible for the gene expression at Start. This transport depends on the specific nuclear localization signals present in cargo proteins.

## Background

In eukaryotic cells, the basic cellular functions occurring inside the nucleus, like transcription or DNA replication, require the transport of proteins across the nuclear envelope. The nucleocytoplasmic transport of most proteins is an energy-dependent process requiring the participation of the transport receptors of the β-karyopherin family [1-7]. β-karyopherins specifically recognize the nuclear localization signals (NLS) or the nuclear export signals (NES) in cargos to carry them through the nuclear pore complex. The binding and release of cargo proteins by karyopherins is controlled by the GTPase Ran cycle [2]. In the import process, karyopherins bind the cargo in the cytoplasm and, once translocated into the nucleus, the binding of Ran-GTP destabilizes the complex releasing the cargo. In the export process, the binding of the cargo by the karyopherins in the nucleus is stabilized by Ran-GTP and, once in the cytoplasm, hydrolysis of GTP to GDP triggers the dissociation of the cargo.

The most prevalent import pathway is the classical import pathway. This pathway transports those proteins carrying classical nuclear localization signals [8]. These signals consist in either a short cluster of basic amino acids (monopartite) or two short clusters of basic amino acids separated by 10-12 residues (bipartite). *S. cerevisiae *Kap95 (homologous to mammalian importin β) is the β-karyopherin involved in the nuclear import of proteins with classical NLS. However, Kap95 is unable to bind the cargo protein directly, but requires an adaptor protein, Srp1 or importin α, to recognize the classical NLS [9-11]. Cse1, another β-karyopherin involved in the classical import pathway, is needed to recycle Srp1 to the cytoplasm once the complex Kap95-Srp1-cargo has been dissociated in the nucleus [12,13]. The *KAP95*, *SRP1 *and *CSE1 *genes are essential for cell viability. A mutation in the *SRP1 *gene arrests cells at the G2/M phase of the cell cycle [11]. This indicates that the classical import pathway plays an essential role in cell cycle progression in mitosis.

A major control point in cell cycle progression in eukaryotic cells occurs at the end of the G1 phase in a process called Start in *Saccharomyces cerevisiae*. In Start, yeast cells decide whether or not to initiate a new cell cycle depending on external (nutrient availability, presence of pheromones) and internal (protein synthesis/cell size, DNA integrity) cues [14]. Execution of Start consists in the activation of a transcriptional program that implies the coordinated expression of a large number of genes [15,16]. A lot of those genes codify for the proteins involved in the events of budding, spindle pole body duplication and DNA replication; that is, the events that are activated at this initial step of the cell cycle.

The gene expression at Start depends on two transcription factors: SBF and MBF [17-19]. Both factors are heterodimeric complexes formed by a common regulatory subunit, Swi6, and two different DNA binding proteins, Swi4 for SBF and Mbp1 for MBF. SBF and MBF show a certain degree of redundancy [20]. Despite this, the SBF- and MBF-depending genes can be clustered into functional categories: the expression of growth, morphogenesis and spindle pole body-related genes, as well as *CLN1 *and *CLN2 *cyclin genes, depend mainly on SBF, while those genes involved in the control and execution of DNA replication and repair, including the *CLB5 *and *CLB6 *cyclin genes, are transcribed by MBF. SBF and MBF bind target promoters at the beginning of G1 [21], but the gene expression is restrained by the association of transcriptional repressor Whi5 [22,23]. At the end of G1, the cyclin-dependent kinase Cdc28-Cln3 enters the nucleus [24] and phosphorylates Whi5, SBF and MBF, thus promoting the dissociation of the repressor and transcriptional activation [22,23,25]. As a result, kinases Cdc28-Cln1 and Cdc28-Cln2 accumulate and act on Whi5, SBF and MBF to establish a positive feedback loop that is important for the proper execution of Start [26]. Later in the cell cycle, this transcriptional wave is inactivated by the phosphorylation of SBF by kinase Cdc28-Clb2 [27] and the association of repressor Nmr1 to MBF [28].

Spatial regulation adds a new step to the control of the protein function. With regards to transcription factors, this spatial regulation necessarily implies a nuclear import mechanism and, in some cases, an export mechanism that could contribute to the control of transcription factor activity. As regards Start transcription factors, Swi4 shows a constant nuclear localization in all the cell cycle stages [29]. However, Swi6 and Whi5 shuttle between the nucleus and the cytoplasm along the cell cycle: they are nuclear from the end of mitosis to the S-G2 phases in the case of Swi6 [30-32] or to Start in the case of Whi5 [22]. Localization of Swi6 is controlled by an import mechanism mediated by a classical NLS [32,33] and an export mechanism that depends on karyopherin Msn5 [31]. Changes in Swi6 localization are basically due to the cell cycle regulation of its nuclear import: phosphorylation of Ser160 by Cdc28-Clb6 blocks the nuclear import during the S-G2 phases until the end of mitosis when the Cdc14 phosphatase removes the phosphate group to once again allow the nuclear accumulation of the protein [30,32,33]. Localization of Whi5 is controlled by an import mechanism mediated by two functionally redundant classical NLS recognized by karyopherin Kap95 and an export mechanism that depends on karyopherin Msn5 [25,34]. Conversely with Swi6, the nuclear import of Whi5 is not cell cycle-regulated, and changes in localization are due to a regulated nuclear export: multiple phosphorylation of specific residues drives the export until this phosphorylation is reverted by the Cdc14 phosphatase at the end of mitosis [34].

In this work we studied the role of Kap95 in cell cycle progression. We show that inactivation of Kap95 arrests cells at two critical cell cycle points: G2/M transition and G1/S transition. The SBF and MBF subunits Swi6, Swi4 and Mbp1 are delocalized in *kap95 *cells. As previously shown for Swi6, Srp1 is also required for the nuclear localization of Swi4 and Mbp1. Thus, the classical nuclear import pathway plays a key role in the control of Start regulators.

## Results

### Kap95 is involved in the Start transition of the cell cycle

**Figure 1:**
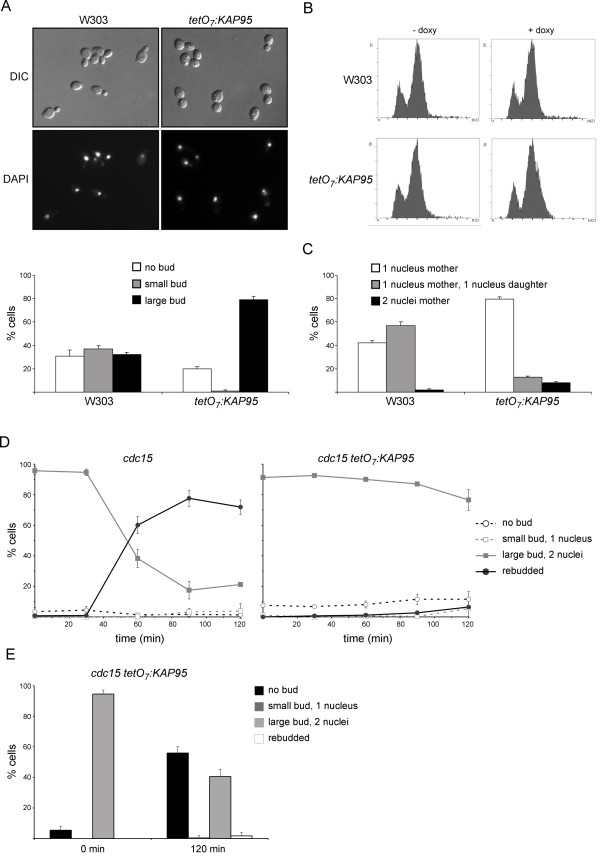
**Characterization of the terminal phenotype of *tetO *_*7*_*:KAP95 *mutant strain**. Exponentially growing cells of the wild type (W303-1a) and *tetO *_*7*_*:KAP95 *(JCY635) strains were incubated in the presence of 5 μg/mL doxycycline for 6 hours. (A) DIC and DAPI staining of DNA images. Graph shows distribution of cells based on the bud size. (B) FACS analysis of DNA content. (C) Distribution of large budded cells based on number and location of nuclei. (D) Exponentially growing cells of the *cdc15 *and *cdc15 tetO *_*7*_*:KAP95 *(JCY1543) strains were incubated in the presence of 5 μg/mL doxycycline at 37° for 4 hours. After transfer to 25°, cell morphology and nuclei number were analyzed at the indicated times (see text). (E) Cell morphology and nuclei number were analyzed in the samples of the *cdc15 tetO *_*7*_*:KAP95 *(JCY1543) strain at 0 and 120 min after digestion of cell wall with zymolyase.

The classical nuclear import pathway is essential for cellular functions since the inactivation of any of its components results in loss of cell viability. Studies with an *srp1 *mutant strain showed that mutant cells arrested at the G2/M transition, which indicates that this pathway plays an essential role at the beginning of mitosis [11]. By considering the important role of the classical nuclear import pathway, we wondered whether its inactivation would affect the regulation of other cell cycle stages. *KAP95 *is an essential gene. In order to investigate its function, we constructed a conditional mutant strain that expresses the *KAP95 *gene under the control of the doxycycline-regulated *tetO_7 _*promoter. As expected, cells were unviable in YPD containing 5 μg/mL doxycyclyne (data not shown). In a first attempt to characterize the function of *KAP95*, doxycycline was added to exponentially growing *tetO *_*7*_*:KAP95 *cells and their terminal phenotype was analyzed after 6 hours. Cell morphology revealed the presence of two populations of cells: approximately 80% of cells presented a large bud whose size was similar to the mother cell; the remaining 20% of cells had no bud (Figure 1A). In agreement with this morphology, the flow cytometry analysis of DNA content showed a major 2N peak and a minor 1N peak in the doxycycline-treated *tetO *_*7*_*:KAP95 *cells. Importantly, inactivation of Kap95 caused a defect blocking entry into the S phase, as deduced from the marked decrease in the valley between the two discrete DNA peaks (Figure 1B). Finally, we analyzed the nuclear morphology in the absence of Kap95. As expected, DAPI staining revealed that *kap95 *unbudded cells contained a single nucleus (Figure 1C). As regards cells with a large bud, conversely to what was observed in the wild-type cultures, most (approximately 80%) of the *kap95 *large budded cells showed a single nucleus, and only 20% of them contained two nuclei. This result indicates a defect in mitosis in the *kap95 *mutant. It is noteworthy that the nucleus in *kap95 *cells is sometimes displaced from the bud neck and that approximately 8% of large budded cells segregate the DNA inside the mother cells, which suggests a defect in nucleus positioning.

In conclusion, all these observations indicate that the inactivation of Kap95 causes a double arrest in cell cycle progression. Most cells accumulate with a large bud, duplicated DNA and a single nucleus, indicating that the absence of Kap95 generates a major arrest in the G2-M transition. This is in agreement with the results previously reported for the mutants in the other karyopherins of the classical import pathway [11,35]. It is noticeable, however, that a minor yet significant number of *kap95 *cells (approximately 20%) accumulated as unbudded cells with unreplicated DNA and a single nucleus, indicating a G1 arrest.

In order to further characterize the defect in executing Start in the absence of Kap95, cells were synchronized at the telophase by means of a thermosensitive *cdc15 *mutant allele. After release from the arrest, progression through Start was monitored by the occurrence of rebudding (*cdc15 *cells show a defect in cytokinesis/cell separation after recovering from telophase arrest). As it can be observed in figure 1D, in the case of the control strain, the rebudded cells accumulated 60 min after release from the arrest, representing approximately 80% of the total cells after 90 min. Conversely, *kap95 *mutant cells failed to rebud after the release, even after 120 min. This was not due to a defect in the recovery from telophase arrest since, unlike what was observed with the telophase-arrested cells, digestion of the cell wall with zymoliase rendered most cells unbudded (Figure 1E). Thus, the lack of rebudding indeed reflects the inability to progress through Start in the absence of Kap95.

It is important to note that a similar result to that described for the *tetO *_*7*_*:KAP95 *mutant strain was obtained with a *tetO *_*7*_*:CSE1 *mutant strain (data not shown), indicating that the Start defect is not a specific phenotype of Kap95 inactivation, but must relate to the complete classical import pathway function. The arrest at the G1/S transition is a novel phenotype for the mutants in this pathway and demonstrates a role of the classical nuclear import pathway in the Start transition.

### Start transcription is impaired in the absence of Kap95

**Figure 2:**
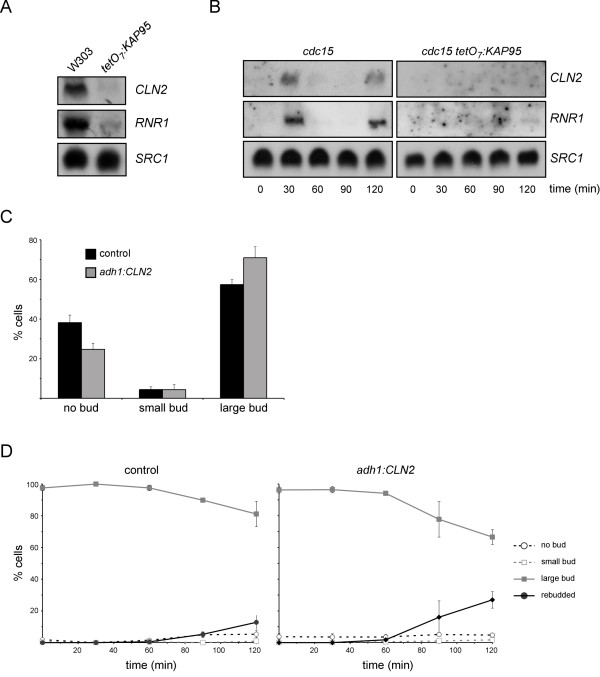
**Analysis of Start transcription in the *kap95 *mutant strain**. (A) Northern analysis of the expression of the Start genes *CLN2 *and *RNR1 *in exponentially growing cells of the wild type (W303-1a) and *tetO *_*7*_*:KAP95 *(JCY635) strains incubated in the presence of 5 μg/mL doxycycline for 6 hours. *SRC1 *transcript is shown as a loading control. (B) Exponentially growing cells of the *cdc15 *and *cdc15 tetO *_*7*_*:KAP95 *(JCY1543) strains were incubated in the presence of 5 μg/mL doxycycline at 37° for 4 hours. After transfer to 25°, expression of *CLN2 *and *RNR1 *genes at the indicated times was analyzed by northern analysis (C) Exponentially growing cells of the *tetO *_*7*_*:KAP95 *(JCY635) strain transformed with a control plasmid (pRS313) or a plasmid expressing the *CLN2 *gene under the control of the *S. pombe adh1 *promoter were incubated in the presence of 5 μg/mL doxycycline for 6 hours. The graph shows the distribution of cells based on the bud size. (D) Exponentially growing cells of the *cdc15 tetO *_*7*_*:KAP95 *(JCY1543) strain transformed with a control (pRS313) or the *adh1:CLN2 *plasmid were incubated in the presence of 5 μg/mL doxycycline at 37° for 4 hours. After transfer to 25°, cell morphology was analyzed at the indicated times.

At the heart of Start there is a transcriptional program mediated by the SBF and MBF transcription factors whose activation is required for cell cycle initiation. We envisaged the possibility of the G1/S arrest observed in the *kap95 *mutant being due to a defect in the activation of the Start gene expression, a process that necessarily involves the nuclear import of proteins. To test this possibility, the expression of a SBF-regulated gene, *CLN2*, and a MBF-regulated gene, *RNR1*, was analyzed by northern analysis. After the inactivation of the *KAP95 *gene, expression of both *CLN2 *and *RNR1 *was absent (Figure 2A). Start transcription was also analyzed in *cdc15*-synchronized cultures. Whereas in *cdc15 *control cells *CLN2 *and *RNR1 *transcripts were detected 30 minutes after release from the arrest, *cdc15kap95 *cells failed to activate *CLN2 *and *RNR1 *gene expression even 120 minutes after the release (Figure 2B). These results confirm that *kap95 *cells do not activate the Start transcriptional program.

It is known that the ectopic expression of *CLN2 *suppresses the lethality of the mutant strains in the Start transcriptional program. When a plasmid expressing *CLN2 *under the control of the *S. pombe adh1 *promoter was introduced into the *tetO *_*7*_*:KAP95 *mutant strain, the percentage of G1 cells significantly lowered: 24% compared to 38% for the control plasmid (Figure 2C). This suggests that the defect in Start transcription contributes to the blockage of *kap95 *mutant cells at G1. However, many G1 cells are still present, which suggest that other functions involved in the G1/S transition are still affected. A similar result was obtained in synchronized *cdc15 tetO *_*7*_*:KAP95 *cultures. As it can be observed in Figure 2D, the ectopic expression of *CLN2 *caused a significant increase in the percentage of rebudded cells 120 minutes after the release from the arrest; however, most of the cells were still unable to initiate a new cell cycle. In conclusion, these results indicate that the impaired Start gene expression contributes to the blockage of *kap95 *mutant cells at G1 but that other affected functions in addition to the transcriptional program cause the defect in cell cycle progression through the G1/S transition.

### Role of Kap95 in Swi6 nuclear import

**Figure 3:**
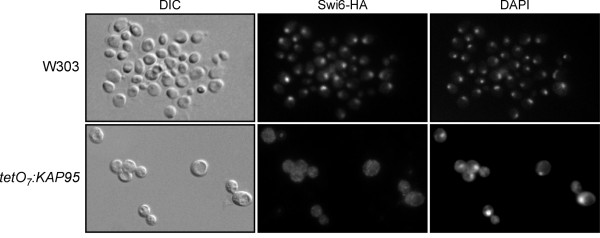
**Subcellular localization of Swi6 in the *kap95 *mutant strain**. Cells from exponentially growing cultures of the wild type (JCY710) and *tetO *_*7*_*:KAP95 *(JCY637) strains expressing the HA-tagged version of Swi6, were incubated in the presence of 5 μg/mL doxycycline for 6 hours and assayed by indirect immunofluorescence. The HA indirect-fluorescence signals, the DAPI staining of DNA and DIC images are shown. No signal was detected in a control of the untagged W303-1a strain.

The previous results led us to study the control of the subcellular localization of the transcriptional machinery of Start by karyopherin Kap95. Swi6 shuttles between the nucleus and the cytoplasm: it enters the nucleus at the end of mitosis until S-G2, when it relocates to the cytoplasm. Swi6 nuclear entry depends on a NLS whose function is regulated by the phosphorylation state of Ser160 [32]. Swi6 NLS interacts *in vitro *with Srp1 and, consistently, Swi6 accumulates in the cytoplasm in the *srp1 *mutant strain [33]. To confirm that Swi6 enters the nucleus via the classical Srp1-Kap95 pathway, we studied the distribution of Swi6 in the doxycycline-treated *tetO:KAP95 *cells by indirect immunofluorescence. Since Swi6 is cytosolic in the G2/M phase, the putative effect of Kap95 inactivation on Swi6 localization must be analyzed in the G1 population of cells when the transcription factor is normally nuclear. As Figure 3 illustrates, Swi6 accumulates in the nucleus of the wild-type G1 cells, but is cytosolic in the *kap95 *G1 cells. This result demonstrates that the Swi6 import in G1 depends on Kap95 and, therefore, on a complete classical import pathway.

### Characterization of Swi4 nuclear import

**Figure 4:**
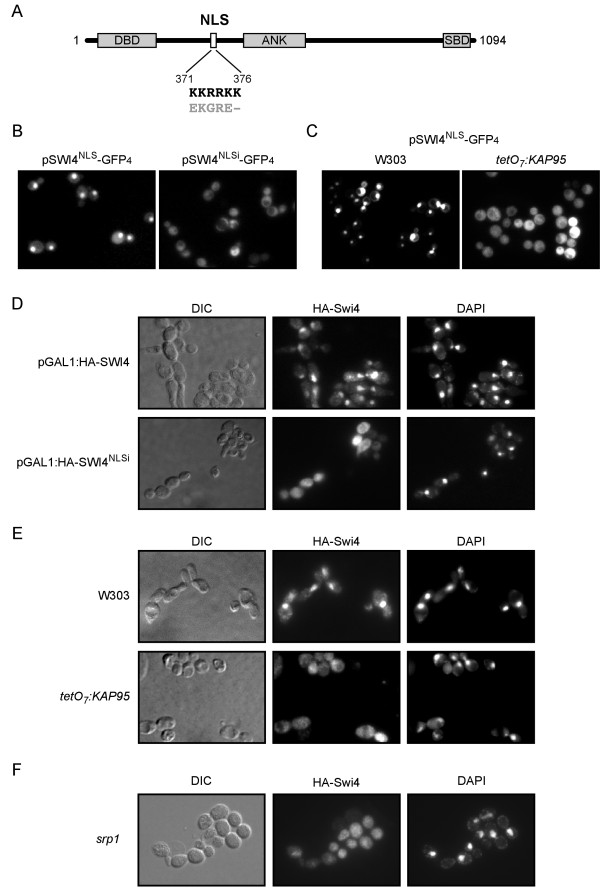
**Control of the subcellular localization of Swi4**. (A) A putative NLS sequence in the Swi4 protein is indicated (white box). Amino acids substitutions introduced to inactivate the NLS are shown in grey. Grey boxes represent the DNA binding (DBD), the ankyrin (ANK) and the Swi6 binding (SBD) domains. (B) Exponentially growing cells of the wild type strain (W303-1a) transformed with plasmids pSWI4^NLS^-GFP_4 _and pSWI4^NLSi^-GFP_4 _were analyzed by fluorescence microscopy. GFP signal images are shown. (C) Exponentially growing cells of the wild type (W303-1a) and *tetO_7_:KAP95 *(JCY635) strains transformed with plasmid pSWI4^NLS^-GFP_4 _were incubated in the presence of 5 μg/mL doxycycline for 6 hours and analyzed by fluorescence microscopy. GFP signal images are shown. (D) Exponentially growing cells of the wild type strain (W303-1a) transformed with plasmids pGAL1:HA-SWI4 and pGAL1:HA-SWI4^NLSi ^were incubated in the presence of 5 μg/mL doxycycline for 6 hours and assayed by indirect immunofluorescence. The HA indirect-fluorescence signals, the DAPI staining of DNA and DIC images are shown. (E) Exponentially growing cells of the the wild type (W303-1a) and *tetO *_*7*_*:KAP95 *(JCY635) strains transformed with plasmid pGAL1:HA-SWI4 were incubated in the presence of 5 μg/mL doxycycline for 6 hours and assayed by indirect immunofluorescence. The HA indirect-fluorescence signals, the DAPI staining of DNA and DIC images are shown. (F) Exponentially growing cells of the the wild type (W303-1a) and the *srp1 *strains transformed with the plasmid pGAL1:HA-SWI4 were incubated at 37° for 2 hours and and assayed by indirect immunofluorescence. The wild type cells showed a nuclear localization of Swi4 similar to that showed in (E) and are not shown.

Swi4, the DNA binding subunit of the SBF transcription factor, has been intensely studied. Nonetheless, little is known about the control of its subcellular localization. Unlike Swi6, Swi4 resides in the nucleus throughout the cell cycle, but neither the karyopherin nor the NLS mediating its nuclear import has been identified. In a first approach, we examined the Swi4 sequence and detected a putative NLS between amino acids 371 and 376 (KKRRKK) that fitted the consensus classical NLS sequence perfectly (Figure 4). The absence of phosphorylable residues in the NLS vicinity suggests that, unlike Swi6 NLS, it would not be regulated, so it would be able to constantly introduce Swi4 into the nucleus. To test the function of this putative NLS, we fused it to four copies of GFP (to avoid free diffusion through the nuclear pore complex). While the control GFP_4 _protein was located in the cytoplasm, the Swi4^NLS^-GFP_4 _chimera showed a nuclear localization throughout the cell cycle, similarly to that observed for the NLS^SV40^-GFP_4 _positive control (Figure 4B and data not shown), which strongly suggests a constant active import of Swi4. In addition, the direct mutagenesis of the basic residues in the NLS sequence led to a cytosolic distribution of the resulting Swi4^NLSi^-GFP_4 _chimera. These results demonstrate that amino acids 371 to 376 of Swi4 constitute a functional NLS which is sufficient to introduce a protein into the nucleus.

The high degree of homology between Swi4 NLS and the consensus classical NLS suggests that the classical import pathway could import the Swi4^NLS^-GFP_4 _protein into the nucleus. To test this dependency, we analyzed the distribution of the Swi4^NLS^-GFP_4 _protein in the absence of Kap95. As observed in Figure 4C, Kap95 inactivation resulted in a cytoplasmic localization of the chimera, which indicates that Kap95 may recognize the NLS from Swi4.

Next we examined the role of the identified NLS in the nuclear import of Swi4 by analyzing the effect of its inactivation on Swi4 localization. As endogenous levels of Swi4 are difficult to detect by indirect immunofluorescence, we expressed Swi4 under the control of the *GAL1 *promoter to increase the amount of protein. The overexpression of Swi4 did not alter the localization of the protein, which accumulated in the nucleus of all the cells (Figure 4D). Nevertheless, inactivation of the described NLS resulted in a cytoplasmic localization of Swi4. Therefore, this NLS is necessary to drive the nuclear localization of Swi4.

Finally, we investigated the localization of Swi4 in the doxycycline-treated *tetO:KAP95 *cells. Consistently with the previous result, inactivation of Kap95 caused a homogeneous Swi4 distribution through the cell (Figure 4E). The same result was observed in a mutant strain in the importin α Srp1 (Figure 4F). All the obtained results help establish the mechanism underlining the nuclear localization of Swi4. Swi4 has a unique NLS between amino acids 371 and 376 which is sufficient and necessary for its nuclear import, an import that is mediated by the classical import pathway involving karyopherins Kap95 and Srp1. Therefore, Swi4 is another Start regulator whose nuclear localization depends on Kap95-Srp1 activity.

### Role of Kap95 in the nuclear import of Mbp1

**Figure 5:**
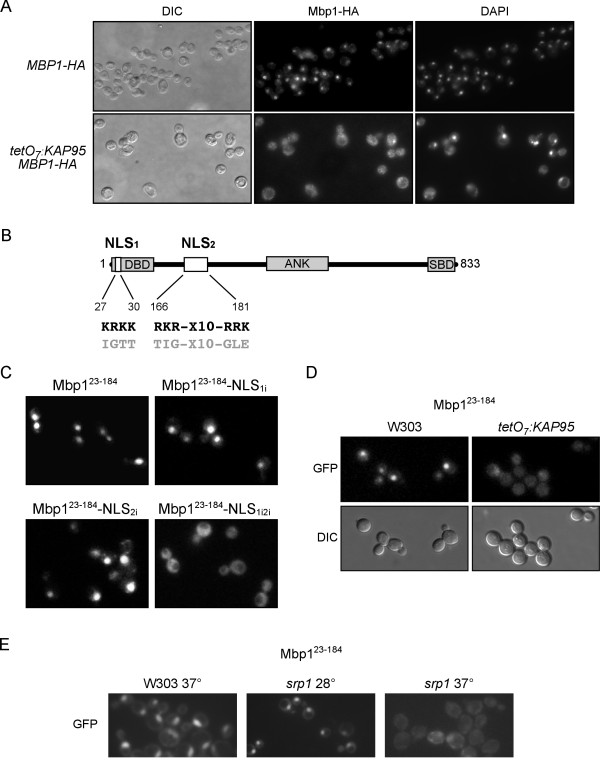
**Control of the subcellular localization of Mbp1**. (A) Cells from exponentially growing cultures of the wild type (JCY816) and *tetO *_*7*_*:KAP95 *(JCY848) strains expressing the HA-tagged version of Mbp1, were incubated in the presence of 5 μg/mL doxycycline for 6 hours and assayed by indirect immunofluorescence. The HA indirect-fluorescence signals, the DAPI staining of DNA and DIC images are shown. (B) Putative NLSs sequences in the Mbp1 protein are indicated as white boxes. Amino acids substitutions introduced to inactivate the NLSs are shown in grey. Grey boxes represent the DNA binding (DBD), the ankyrin (ANK) and the Swi6 binding (SBD) domains. (C) Exponentially growing cells of the wild type strain (W303-1a) transformed with plasmids pMBP1^23-184^-GFP4, pMBP1^23-184NLS1i^-GFP4, pMBP1^23-184NLS2i^-GFP4 and pMBP1^23-184NLS1i2i^-GFP4 were analyzed by fluorescence microscopy. GFP signal images are shown. (D) Exponentially growing cells of of the wild type (W303-1a) and *tetO_7_:KAP95 *(JCY635) strains transformed with plasmid pMBP1^23-184^-GFP4 were incubated in the presence of 5 μg/mL doxycycline for 6 hours and analyzed by fluorescence microscopy. GFP signal and DIC images are shown. (E) Exponentially growing cells of the the wild type (W303-1a) and the *srp1 *strains transformed with plasmid pMBP1^23-184^-GFP4 were incubated at 37° for 2 hours. GFP signal images are shown.

Mbp1 is the DNA binding protein of the Start transcription factor MBF. Despite being important for Start, there is no data about the subcellular localization of Mbp1. In a first approach, the cellular distribution of an HA-tagged Mbp1 protein expressed at the endogenous level was analyzed by indirect immunofluorescence to investigate whether the subcellular localization of Mbp1 changes along the cell cycle. As Figure 5 depicts, Mbp1 fluorescence colocalized with the DAPI signal in cells in all the cell cycle stages. This result demonstrates that under physiological conditions Mbp1 is nuclear throughout the cell cycle.

Next we studied the karyopherin involved in the nuclear import of Mbp1. By considering that Mbp1 and Swi4 belong to the same family of transcription factors, and the fact that they share structural similarities, we envisaged the possibility of them possibly entering the nucleus assisted by the same import route. Therefore, we analyzed Mbp1 distribution in a mutant in karyopherin Kap95. The immunofluorescence assays show a cytoplasmic accumulation of Mbp1 when Kap95 was inactivated, unlike the strong nuclear signal observed in the wild-type cells (Figure 5). This result indicates that Mbp1 enters the nucleus assisted mainly by the classical import pathway. Nevertheless, a faint Mbp1 nuclear signal was observed in some *kap95 *cells, suggesting that other karyopherins might contribute to the Mbp1 nuclear import to a lesser extent.

The fact that the nuclear import depends on Kap95 led us to screen the Mbp1 protein for the putative classical NLS sequences. Two putative classical NLSs were identified in the N-terminus region of Mbp1: a monopartite one (NLS1) between amino acids 27 and 30 and a bipartite one (NLS2) located between amino acids 166 and 181 (Figure 5B). In order to test whether these putative NLSs were in fact functional, we fused a fragment of Mbp1 from amino acids 23 to 184 (containing both candidate NLSs) to four copies of GFP. Unlike the control GFP_4 _protein, the chimera Mbp1^23-184^-GFP_4 _localized in the nucleus (Figure 5C), thus demonstrating the existence of at least one functional NLS in this region of the Mbp1 protein. As expected, the Mbp1^23-184^-GFP_4 _protein became cytosolic when Kap95 was inactivated (Figure 5D). Importantly, the nuclear accumulation of Mbp1^23-184^-GFP_4 _in a *srp1 *mutant strain was also eliminated (Figure 5E). These results confirm that the nuclear import activity in fragment 23-184 of Mbp1 requires an intact classical import pathway

Further characterization of Mbp1 NLS was carried out by a direct mutagenesis of the basic residues in both putative NLSs, as indicated in Figure 5B. The subcellular localization of the resulting Mbp1^23-184-NLS1i^-GFP_4 _and Mbp1^23-184-NLS2i^-GFP_4 _chimeras was analyzed. In both cases, inactivation of a single putative NLS did not significantly change the location of the proteins, which were clearly detected in the nucleus. Thus, neither of the two putative NLSs is essential for the nuclear import of Mbp1. Importantly, however, when both putative NLSs were simultaneously mutated, nuclear localization was avoided and the Mbp1^23-184-NLS1i2i^-GFP_4 _protein was located mainly in the cytoplasm. These results demonstrate that Mbp1 contains two functional and redundant NLSs, and that either of them is able to mediate the nuclear import of Mbp1 by Srp1-Kap95 karyopherins.

## Discussion

Two of the first karyopherins to be connected to cell cycle regulation were Srp1 and Cse1, which together with Kap95, constitute the classical nuclear import system of proteins. The inactivation of Srp1, and to a lesser extent the inactivation of Cse1, causes an arrest in cell cycle progression at the G2/M phase [11,35]. Consistently, in this work we describe how the inactivation of Kap95 triggers a blockage at the G2/M transition. However, the inactivation of Kap95 also originates a previously uncharacterized accumulation of cells in G1. This result could indicate that Kap95 may undertake a function in G1/S that is not shared with Srp1. To support this possibility, Kap95 has been recently described to contribute to the import of proteins without requiring Srp1 and by recognizing an import signal distinct from the classical NLS [36,37]. However, the fact that the major cell cycle defect in the *kap95 *mutant also occurs in G2/M and, more importantly, the fact that we also observed G1 arrested cells in a *tetO *_*7*_*:CSE1 *mutant strain, suggest that the different phenotype we observed in the *kap95 *mutant cells, compared to the previously reported phenotype of mutations *srp1-31 *and *cse1-1*, is more likely caused by the different effect of the distinct mutant used in the classical nuclear import pathway activity. In fact, the nuclear localization of the key Start regulators described in this work depends on both Kap95 and Srp1. Thus, our results reveal a new function of the classical import pathway in the control of the cell cycle at the Start transition. Nevertheless, it cannot be ruled out that Kap95 might control other proteins involved in the G1/S transition independently of Srp1. The fact that only a minor proportion of *kap95 *mutant cells are blocked in G1 could simply reflect a greater sensitivity of the G2/M transition to the reduced activity of the pathway, so the blockage of cells at this cell cycle step precludes an accumulation of cells in the G1 phase. This is supported by the results obtained when cells were arrested at the telophase (after the major G2/M arrest point) before inactivating *KAP95*. In this case, cells mostly accumulate in the G1 phase, demonstrating that Kap95 is required for executing Start.

Despite the defect in the G2/M phase in the mutants in the classical import pathway having been characterized several years ago, there is no explanation for the causes of this arrest. As inactivation of Srp1 results in longer half lives of some mitosis regulators like cyclin Clb2, some authors have proposed that this import pathway could affect the protein degradation machinery required for progression through mitosis [11]. Alternatively, the inability to enter the nucleus of a key mitotic regulator required for mitosis progression could also explain the blockage at the G2/M transition. A good candidate is separase Esp1, the protease that triggers sister-chromatid segregation once in the nucleus [38,39]. Failure to locate separase in the nucleus is expected to result in cell arrest at the metaphase, which is consistent with the terminal phenotype of the mutants in the classical import pathway. Interestingly, the analysis of the Esp1 sequence identified a consensus classical monopartite NLS between amino acids 1188 and 1192 (RRKRR), which strongly suggests that the classical nuclear import pathway may be responsible for separase nuclear accumulation.

It is noteworthy that additional defects have been observed in the G2/M *kap95 *cells. First, most G2/M cells contain a single nucleus, although this is not properly located near the bud neck in a fraction of the cells. Second, a small yet significant percentage of cells that escapes the blockage segregates chromosomes inside mother cells. A similar defect was observed in the *cse1-1 *mutant strain [35]. These observations suggest that the inactivation of the classical import pathway causes a dysfunction in the nucleus positioning and/or spindle orientation.

As previously mentioned, our results reveal a new function of karyopherin Kap95 and the classical import pathway in the control of the cell cycle in addition to its known connection with mitosis. Specifically, Kap95 is involved in the execution of Start at the initiation of a new round of division, which suggests that the classical import pathway may mediate the nuclear import of proteins controlling Start. Start consists in the activation of a transcriptional program, so the transcription factors involved in Start are good candidates to be regulated by Kap95. In fact, the expression of G1/S regulated genes is impaired in the absence of Kap95. Given the robustness of the Start transcriptional program conferred by the functional redundancy between its components, we suspected that the G1 arrest in the absence of Kap95 could reflect the delocalization of several Start regulators. This was indeed the case since transcription factors Swi6, Swi4 and Mbp1 enter the nucleus assisted by Kap95 and Srp1. It is important to note that the ectopic expression of *CLN2 *does not completely suppress the G1/S arrest of the *kap95 *mutant cells. This indicates that Kap95 must control the additional proteins besides the Start transcriptional activators, whose mislocalization in *kap95 *mutant cells could contribute to the blockage of the Start transition. As regards Swi6 and Swi4, Kap95 is the only importin responsible for their nuclear localization. Regarding Mbp1, Kap95 is the main import pathway; however, nuclear accumulation in the *kap95 *mutant strain is not completely abolished. This could be interpreted by the presence of a residual Kap95 activity in the cells that are able to import Mbp1, but not Swi4 or Swi6. However, the fact that the overexpression of an Mbp1 protein in which the two classical NLSs are inactivated still shows significant nuclear localization (data not shown), points to the existence of a secondary import pathway besides Kap95. To characterize this alternative pathway, we analyzed the distribution of Mbp1^NLS1i2i ^in all the β-karyopherin mutants (except *kap123*), but the nuclear localization of Mbp1 was not completely abolished in any of the mutant cells (data not shown). This strongly suggests that several alternative minor import pathways are able to translocate Mbp1 into the nucleus. The presence of multiple import pathways for Mbp1 is not unique between yeast proteins. Several cases of proteins imported by several β-karyopherins have been described, such as Sas2, the histones H3 and H4, ribosomal proteins and ribosomal associated proteins, which enter the nucleus via Pse1 and Kap123 [40-43]. There is also the more dramatic case of the HO endonuclease [44] and the Asr1 protein [36], whose nuclear import could be assisted by four or five different importins, respectively.

Besides being involved in the import of Start transcription activators, we have recently shown that Kap95 is the importin of the Start transcriptional repressor Whi5 [34]. By taking into account that the nuclear import of Cln3, the cyclin that initiates the Start transcription program when it is translocated to the nucleus, depends on a canonical classical NLS [45,46], it is conceivable that Cln3 might also be imported by the classical import pathway. Thus, it is striking that the nuclear import of the complete transcriptional machinery of the Start transition is mediated by the classical import pathway. The use of the same pathway should prove advantageous for the coordinated assembling of the transcriptional circuitry. This is particularly relevant when transcriptional activators and repressors restraining its activity exist. The fact that the components of SBF, MBF and Whi5 enter the nucleus by Kap95 facilitates the colocalization of activators with its repressor. If each regulator entered the nucleus by distinct import pathways, the system could be more prone to unscheduled transcription and a deregulation of the G1/S transition with the detrimental consequence to cell physiology.

## Conclusions

**Figure 6:**
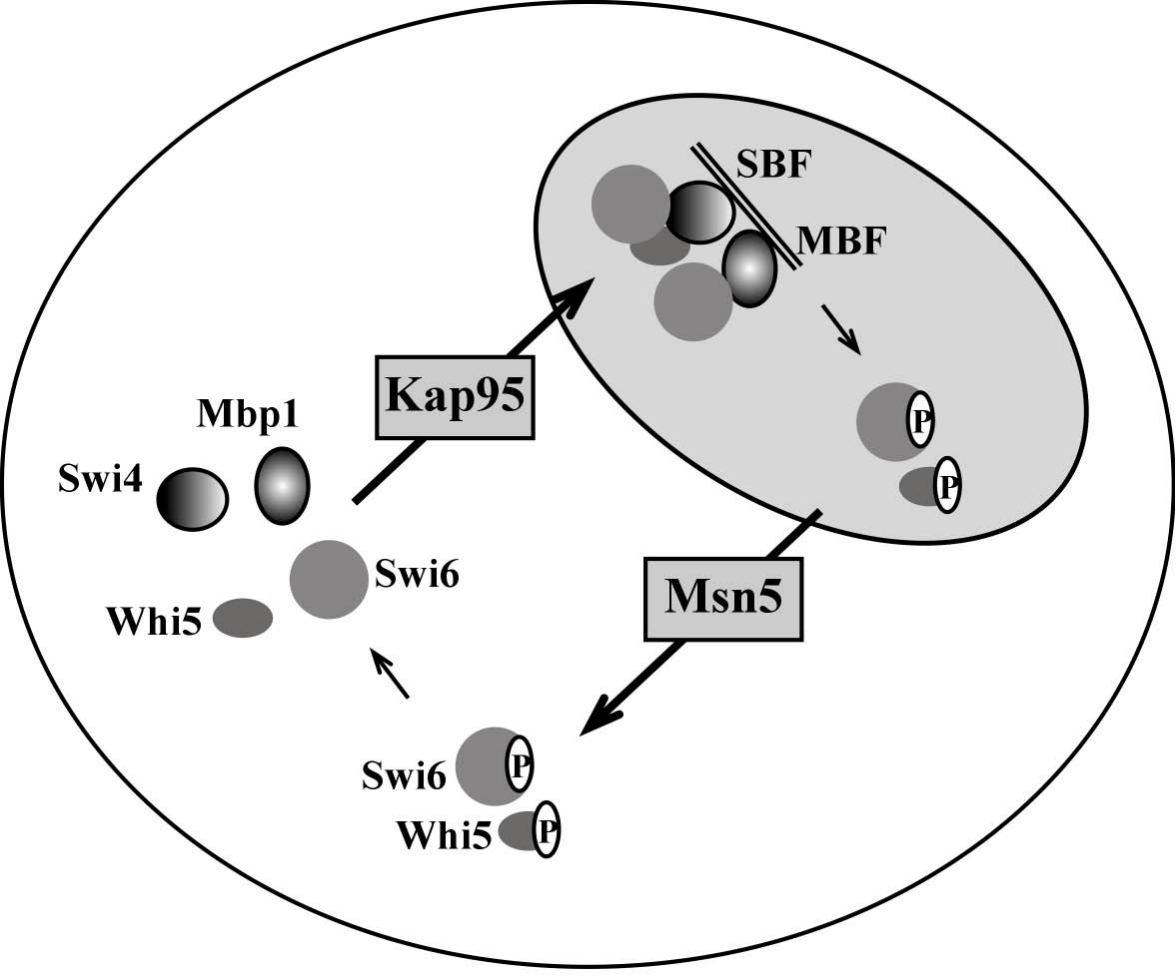
**Coordinated spatial regulation of Start transcriptional machinery**. Expression of genes at the G1/S transition is regulated by the transcription factors SBF (Swi4-Swi6), MBF (Mbp1-Swi6) and the transcriptional repressor Whi5. The four proteins Swi4, Swi6, Mbp1 and Whi5 are imported into the nucleus by the same pathway involving karyopherin Kap95 and classical NLS sequences. Swi4 and Mbp1 remains nuclear along the cell cycle. However, after Start activation Whi5 and Swi6 are exported to the cytoplasm by karyopherin Msn5, a transport that depends on the phosphorylation of the proteins. At the end of mitosis, the Cdc14 phosphatase dephoshorylates Whi5 and Swi6 to once again lead to the nuclear acumulation of the proteins.

Our results reveal a new function of karyopherin Kap95 in the control of the cell cycle. In addition to its known connection with mitosis, Kap95 is required for the execution of Start at the initiation of a cell cycle. This suggests that Kap95 may mediate the nuclear import of proteins controlling Start. We have demonstrated that transcription factors Swi6, Swi4 and Mbp1, responsible for the regulated gene expression at the beginning of the cell cycle, enter the nucleus assisted by Kap95. The nuclear import of Swi4 and Mbp1 (as previously shown for Swi6) is also dependent on importin α Srp1. The nuclear localization signals in Swi4 and Mbp1 mediating the nuclear import of the proteins have been identified. The results described herein complete a detailed picture of the spatial regulation of the components of the essential Start transcriptional program (Figure 6).

## Methods

### Yeast strains and plasmids

Strains used in this study are W303-1a (*MATa leu2 ade2 trp1 his3 ura3 can1 ssd1-d)*, *cdc15 *(*MATa leu2 trp1 his3 ura3 cdc15-2)*, *srp1 *(*srp1-31 *in W303-1a), JCY635 (*tetO *_*7*_:*KAP95-KanMX6 *in W303-1a), JCY710 (*SWI6-HA-TRP1 *in W303-1a), JCY637 (*SWI6-HA-TRP1 *in JCY635), JCY816 (*MBP1-HA-TRP1 *in W303-1a), JCY817 (*TRP1*-*GAL1:HA-SWI4 *in W303-1a), JCY848 (*MBP1-HA-TRP1 *in JCY635) and JCY1543 (*tetO*_*7*_:*KAP95-KanMX6, CMVp(tetR'-SSN6)::LEU2 *in *cdc15*). The substitution of the *KAP95 *promoter by the *tetO*_*7 *_promoter was obtained by integrating a DNA fragment amplified from plasmid pCM225 (a gift from E. Herrero). The susbtitution of the SWI4 pormotoer by the GAL1 promoter was obtained by integrating a DNA fragment amplified from plasmid pFA6a-TRP1-PGAL1-3HA (from Dr. J.R. Pringle). Tagging of the Swi6 and Mbp1 protein at the C-terminus with HA was achieved by integrating a DNA fragment amplified from plasmid pF6a-HA:TRP1 (from Dr. J.R. Pringle). Yeast cells were grown on standard yeast extract-peptone-dextrose (YPD) media or synthetic dextrose (SD) or galactose (SR) minimal media supplemented as required. To repress the *tetO*_*7 *_promoter, doxycycline was added to a concentration of 5 μg/ml.

Plasmid pSWI4^NLS^-GFP_4 _derived from pNLS^SV40^-GFP_4 _[34], which contains, in order, the *ADH1 *promoter, the SV40 NLS, and four *GFP *gene copies). It was obtained by site directed mutagenesis resulting in the substitution of the SV40 NLS by the SWI4 NLS (KKRRKK). pSWI4^NLSi^-GFP_4 _plasmid was obtained from pSWI4^NLS^-GFP_4 _by site directed mutagenesis of the SWI4 NLS to an inactive NLS version (EKGRE).

Plasmid pMBP1^23-184^-GFP_4 _was obtained removing the SV40 NLS coding region from pNLS^SV40^-GFP_4 _by KpnI-BamHI digestion and introducing an *MBP1 *gene fragment coding from amino acid 23 to 184 amplified from genome with a forward oligo containing a KpnI restriction site and a reverse oligo containing a BamHI site. pMBP1^23-184 NLS1i^-GFP_4 _plasmid was obtained from pMBP1^23-184^-GFP_4 _by site directed mutagenesis of Mbp1 NLS1 (KRKK) to an inactive NLS version (IGTT). pMBP1^23-184 NLS2i^-GFP_4 _plasmid was obtained from pMBP1^23-184^-GFP_4 _by site directed mutagenesis of Mbp1 NLS2 (RKR-X_10_-RRK) to an inactive NLS version (TIG-X_10_-GLE). pMBP1^23-184 NLS1i2i^-GFP_4 _plasmid was obtained from pMBP1^23-184 NLS2i^-GFP4 by site directed mutagenesis of Mbp1 NLS1 (KRKK) to an inactive NLS version (IGTT).

pGAL1:HA-SWI4 plasmid was obtained by cloning a DNA fragment containing the coding region for an HA N-terminal tagged Swi4 protein expressed under the control of *GAL1 *promoter, amplified from the genome of JCY817 using a forward oligo containing a SalI restriction site and a reverse oligo containing a KpnI site in SalI-KpnI digested YCplac33 vector. pGAL1:HA-SWI4^NLS1i ^plasmid was obtained from pGAL1:HA-SWI4 by site directed mutagenesis of the SWI4 NLS (KKRRKK) to an inactive NLS version (EKGRE).

### DNA content analysis

Approximately 10^7 ^cells were fixed in EtOH 70%. Fixed cells were treated with RNase A (1 mg/mL) in Tris-HCl 50 mM [pH 7.5] buffer for 3 hours at 37°C and pepsine 5 mg/mL for 5 minutes at room temperature. Finally cells were stained with propidium iodure (50 μg/mL). DNA content was analyzed in a EPICS XL (Coulter Inc.) cytometer.

### Fluorescent microscopy

For indirect immunofluorescence assays, approximately 10^8 ^cells were fixed in growth medium with 37% formaldehyde (1:10 dilution) for 60 min with agitation. After mild sonication, cells were collected, washed with buffer A (1.2 M sorbitol, 0.1% β-mercaptoethanol, 50 mM KH_2_PO_4 _[pH 7.0]), and incubated at 37°C for 1 to 2 h in 0.5 ml of buffer A containing 100 μg of zimolyase 20-T/ml. Spheroplasts were collected, washed with buffer A, resuspended in 20 μl of a solution (8 μg/ml of rat anti-HA antibody 3F10 from Roche in phosphate-buffered saline, PBS, containing 0.1% bovine serum albumin), and incubated overnight at 4°C. After washing with PBS, PBS containing 0.1% NP-40, and PBS, cells were incubated with the secondary antibody Alexa 546-labeled anti-rat IgG antibody (Molecular Probes) as indicated for the primary antibody. DNA was stained at the end of the process by resuspending spheroplasts in PBS containing 1 μg/ml of DAPI (4,6-diamidino-2-phenylindole, Sigma Inc.). In the case of Mbp1-HA immunofluorescence assays, an additional step was included in order to amplify the signal: after incubation with the primary rat anti-HA antibody 3F10, cells were sequentially incubated with Alexa 546-labeled goat anti-rat IgG antibody and Alexa 546-labeled rabbit anti-goat IgG antibody (Molecular Probes).

GFP tagged proteins were visualized in living cells grown in synthetic media. Confirmation of GFP signal as a nuclear signal was achieved in control experiments by DAPI staining of absolute ethanol fixed cells. All microscope samples were analyzed in an *Axioskop 2 *microscope (*Zeiss Inc.*). Images were captured with a *SPOT *camera (*Diagnostic Instruments Inc*.).

## Authors' contributions

FJT carried out the experiments, analyzed the data, wrote the Methods section and revised the manuscript. J.C.I. planned, supervised, and analyzed the data and wrote the manuscript. All authors read and approved the final manuscript.
